# Comparing SNP panels and statistical methods for estimating genomic breed composition of individual animals in ten cattle breeds

**DOI:** 10.1186/s12863-018-0654-3

**Published:** 2018-08-09

**Authors:** Jun He, Yage Guo, Jiaqi Xu, Hao Li, Anna Fuller, Richard G. Tait, Xiao-Lin Wu, Stewart Bauck

**Affiliations:** 1 Biostatistics and Bioinformatics, Neogen GeneSeek Operations, Lincoln, NE USA; 2 College of Animal Science and Technology, Hunan Agricultural University, Changsha, China; 3 College of Education and Human Sciences, University of Nebraska, Lincoln, NE USA; 4 Department of Statistics, University of Nebraska, Lincoln, NE USA; 5 Department of Animal Sciences, University of Wisconsin, Madison, WI USA

**Keywords:** Cattle, Genomic breed composition, Genomic prediction, Admixture model, SNP

## Abstract

**Background:**

SNPs are informative to estimate genomic breed composition (GBC) of individual animals, but selected SNPs for this purpose were not made available in the commercial bovine SNP chips prior to the present study. The primary objective of the present study was to select five common SNP panels for estimating GBC of individual animals initially involving 10 cattle breeds (two dairy breeds and eight beef breeds). The performance of the five common SNP panels was evaluated based on admixture model and linear regression model, respectively. Finally, the downstream implication of GBC on genomic prediction accuracies was investigated and discussed in a Santa Gertrudis cattle population.

**Results:**

There were 15,708 common SNPs across five currently-available commercial bovine SNP chips. From this set, four subsets (1,000, 3,000, 5,000, and 10,000 SNPs) were selected by maximizing average Euclidean distance (AED) of SNP allelic frequencies among the ten cattle breeds. For 198 animals presented as Akaushi, estimated GBC of the Akaushi breed (GBCA) based on the admixture model agreed very well among the five SNP panels, identifying 166 animals with GBCA = 1. Using the same SNP panels, the linear regression approach reported fewer animals with GBCA = 1. Nevertheless, estimated GBCA using both models were highly correlated (*r* = 0.953 to 0.992). In the genomic prediction of a Santa Gertrudis population (and crosses), the results showed that the predictability of molecular breeding values using SNP effects obtained from 1,225 animals with no less than 0.90 GBC of Santa Gertrudis (GBCSG) decreased on crossbred animals with lower GBCSG.

**Conclusions:**

Of the two statistical models used to compute GBC, the admixture model gave more consistent results among the five selected SNP panels than the linear regression model. The availability of these common SNP panels facilitates identification and estimation of breed compositions using currently-available bovine SNP chips. In view of utility, the 1 K panel is the most cost effective and it is convenient to be included as add-on content in future development of bovine SNP chips, whereas the 10 K and 16 K SNP panels can be more resourceful if used independently for imputation to intermediate or high-density genotypes.

**Electronic supplementary material:**

The online version of this article (10.1186/s12863-018-0654-3) contains supplementary material, which is available to authorized users.

## Background

Estimation of breed identification or composition is very useful in a variety of situations. In farm animals, for example, breed registries are used to record and maintain pedigrees of animals with certain conformational, performance and coat color characteristics that are approved for registry within that breed [1]. For cross-bred animals, knowing the admixture proportions of an individual is helpful to estimate heterozygosity, understand the breeding history of the population to which an animal belongs, and make management decisions for crossbreeding programs [2, 3]. In research, breed information is utilized for quality control of samples, including verification of sample breed identifications and exclusion of samples that do not belong to specific breeds. Validation of genetic relationships of individuals is crucial to control the rate of false associations in disease association studies by avoiding or correcting for population stratifications [4, 5].

Genomic selection has emerged as a powerful tool for genetic improvement of farm animals [6]. Genomic selection is desirable for early in life selection and selection on traits which are difficult or expensive to measure. So far, genomic evaluations primarily have been conducted within breeds [7, 8], but cross-breed evaluations have also been addressed [9, 10]. In the US Holstein evaluation, for example, breed check markers are used to validate animals of Holstein breed, excluding crossbred animals because the genomic prediction system developed in purebred Holstein animals does not provide sufficient genomic prediction accuracy in crossbred animals [11]. On the other hand, there has been work indicating that prediction of crossbred genomic merit could be improved by calculating direct genomic values according to weighted SNP effects from each of the contributing breeds, with the weights of SNP effects being each animal’s genomic composition of these breeds [12].

Ancestry and breed origins were historically estimated using microsatellite markers [13, 14] and recently using SNPs [15–17] and sequence data [18, 19]. Arguably, DNA markers are accurate to estimate genomic breed composition (GBC) of animals because they are capable of measuring realized parental contributions at the genomic level [20] and therefore can help correct pedigree errors and even estimate kinships when pedigree data are incomplete or missing [12]. From a genetic perspective, animal breeds differ in SNP allele frequencies at hundreds and thousands of loci due to domestication, selection, and genetic drift [21]. In reality, an animal breed was formed through either natural adaptation to the environment, selective breeding, or a combination of the two, and each breed has its unique genetic features and therefore appearance (phenotypes), behavior, and/or other characteristics that distinguish it from other breeds. Through SNP genotyping, for example, individual animals can be grouped into genetic clusters (breeds) according to their patterns of multiple-loci genotypes (or haplotypes). For individuals whose ancestors originated in different populations, and those which are admixed, their genetic composition exhibits multiple ancestries associated with multiple different genetic clusters or populations, which can be described by admixture models [22–24]. Alternatively, GBC can be estimated using a linear regression model, in which discrete random variables corresponding to counts of certain alleles of reference SNPs across the genome are regressed on the allele frequencies of each reference SNP in a number of known breeds [15]. This latter approach has been used to estimate breed composition in pigs [1] and cattle [12, 17].

In the present study, five SNP panels were derived for estimating GBC amongst 10 cattle breeds, which consisted of 1,000 (1 K), 3,000 (3 K), 5,000 (5 K), 10,000 (10 K), and 15,708 (16 K) SNPs, respectively. More breeds will be included as their genotype data become available. The 16 K consisted of all common, informative SNPs across five historical and currently available SNP chips, and the remaining four panels were selected subsets from 16 K by maximizing the average Euclidean distance of allele frequencies among the ten cattle breeds. With these five selected SNP panels, their comparative performance in estimation of GBC was evaluated in an Akaushi population, based on two statistical models (namely, admixture and linear regression). Finally, downstream implication on genomic selection accuracies was investigated in a population of purebred and crossbred Santa Gertrudis cattle by calibrating SNP effects only on 1,225 Santa Gertrudis cattle with GBC of Santa Gertrudis (GBCSG) being equal to greater than 0.90 and validated on this set of animals and on two sets with lower GBCSG.

## Methods

### Genotype data, reference SNPs, and reference animals

#### Genotype data

The datasets included a total of 29,609 animals of ten cattle breeds, each genotyped on the GeneSeek Genomic Profiler (GGP) bovine 50 K version 1 SNP chip (49,463 SNPs) or GGP LD version 4 SNP chip (40,660 SNPs) (Neogen GeneSeek Operations, Lincoln, NE). Approximately 53% of the animals were from two dairy breeds (Holstein and Jersey) and the remaining 47% were from eight beef cattle breeds (Akaushi, Angus, Beefmaster, Red Angus, Brangus, Hereford, Santa Gertrudis, and Wagyu). Among the beef breeds, Akaushi (Japanese Brown) cattle and Wagyu (Japanese Black) cattle were originally developed in Japan and are well known for their meat quality [25]; Beefmaster was developed in the early 1930s by crossing Hereford cows and Shorthorn cows with Brahman bulls [26]; Santa Gertrudis cattle are a beef breed developed in southern Texas, USA, by mating Brahman bulls with beef Shorthorn cows, with the final composition being about three-eighths Brahman and five-eighths Shorthorn [27]. Average minor allele frequencies (MAF) of all SNPs on the genotyping SNP chip platform across these ten populations varied from 0.188 (Wagyu) to 0.305 (Beefmaster). Descriptive statistics of these genotype data by breed are shown in Table 1. Overall, the accuracy of estimated allele frequencies increased with the sample size. Of the ten breeds, some had large sample size, such as Holstein and Jersey cattle, but the sample sizes for some breeds (such as Akaushi and Santa Gertrudis) were relatively small. For the three composite breeds (Brangus, Beefmaster, and Santa Gertrudis), two of their founder breeds (Brahman and Shorthorn) of the composite cattle were not included in the reference breeds, because genotypes for those breeds were not available at the time at the time of this study.

**Table 1:** Descriptive statistics of genotype data for 29,609 animals used in the present study

Cattle Breed	Number of Genotyped Animals ^a^	Number of SNPs	Average MAF Mean (SD)	Breed Type
Holstein	8,905 (8,863)	49,463	0.295 (0.152)	Dairy
Jersey	6,911 (6,860)	49,463	0.256 (0.158)	Dairy
Akaushi	198 (167)	49,463	0.243 (0.158)	Beef
Angus	4,713 (4,672)	49,463	0.303 (0.152)	Beef
BM	608 (583)	49,463	0.305 (0.142)	Beef
Brangus	1,819 (1,770)	40,660	0.238 (0.161)	Beef
Hereford	2,423 (2,412)	49,463	0.270 (0.150)	Beef
RA	2,229 (2,158)	49,463	0.300 (0.151)	Beef
SG	297 (291)	49,463	0.301 (0.140)	Beef
Wagyu	1,506 (1,506)	40,660	0.188 (0.164)	Beef

*BM* Beefmaster, *RA* Red Angus, *SG* Santa Gertrudis, *MAF* minor allele frequency, *SD* standard deviation of MAF

^a^In the brackets are the number of animals in the reference set for each breed, after removing outliers

#### Selection of reference SNPs

Five panels of reference SNPs were made available. Each panel consisted of common SNPs across five historical or currently-used commercial bovine SNP chips, namely, Illumina Bovine HD (777 K) chip, GGP UHD (150 K) SNP chip, GGP HD (80 K) SNP chip, GGP 50 K version 1 SNP chip, and GGP LD version 4 (40 K) SNP chip. Hulsegge et al. (2013) compared three statistics as the criteria for selecting SNPs [16]: 1) delta (the absolute allele frequency difference between two populations), 2) Wright’s FST, 3) and Weir and Cockerham’s FST. The results of Hulsegge et al. (2013) showed very small differences amongst these three statistics. In the present study, we used average Euclidean distance (AED) of allele frequencies among the breeds, which was equivalent to delta when measured on a single SNP involving only two populations, though mathematically formulated differently. With the number of populations (*T*) &gt; 2, AED was calculated by the Pythagorean formula and then averaged across all possibly unique breed pairs,

1 $$ {AED}_k=\frac{1}{\left(\begin{array}{c}2\\ {}T\end{array}\right)}\sqrt{\sum_{j=1}^T{\sum}_{j\hbox{'}\kern0.36em >j}^T{\left({f}_{jk}-{f}_{j\hbox{'}k}\right)}^2} $$

where *f*_*jk*_ is the frequency of an allele of the *k*-th SNP in the *j*-th breed, and $$ \left(\begin{array}{c}2\\ {}T\end{array}\right) $$ indicates all unique pairs of combinations of the *T* breeds taken 2 breeds at a time without repetition. Note that *f*_*jk*_ can refer to either allele, but it needs to be used consistently. In the present study it refers to the second allele. For example, if SNP genotypes are coded as 0 (AA), 1 (AB) and 2 (BB), then *f*_*jk*_ refers to the frequency of allele B.

Prior to SNP selection, there were 15,708 SNPs (identified as 16 K) in common across the five commercial bovine SNP chips evaluated in this study. The 16 K SNP set are not random, but initially taken as the common set from which four subsets of SNPs, namely 1 K, 3 K, 5 K, and 10 K SNPs, were selected. The SNPs for each subset were selected by maximizing AED of SNP allelic frequencies among the ten breeds, given their respective panel sizes.

#### Selection of reference animals

In the present study, reference animals for each breed were selected using the 1 K SNP panel because the model is parsimonious and the results were very similar across each of the five SNP panels. The likelihood that an animal belonged to a specific breed was computed assuming independent multinomial distributions of its genotypes of these SNPs. Consider one SNP locus with three genotypes, and denote *f*_*kj*(*g*)_ to be the frequency of animals having genotype *g*, where *g* = AA, AB, or BB, respectively, of SNP *k* in the *j*-th population. Let *x* be a genotype of SNP *k* observed on animal *i*. Then, based this SNP only, the likelihood that this animal is a member of population *j* is given by:

2 $$ {L}_{ijk}={\prod}_{g= AA, AB, BB}\left({f}_{jk(x)}^{1_{x=g}}\right) $$

where 1_*x* = *g*_ is an indicator variable, which has a value of 1 if *x* = *g*, or 0 otherwise.

For instance, let this animal have AA genotype for SNP *k*. Then, formula (2) is computed to be:

3 $$ {L}_{ijk}=\left({\left({f}_{AA}\right)}^1\times {\left({f}_{AB}\right)}^0\times {\left({f}_{BB}\right)}^0\right)={f}_{AA} $$

Thus, when only one SNP is considered, the probability that an individual animal belongs to a certain breed, given its observed genotype of this SNP, is equal to the frequency of that genotype in the reference population of that breed.

Now, consider *k* = 1, 2, …, *M* SNPs and let ${l}_{ij}=\left(-2\right)\frac{1}{M}\log {\prod}_{k=1}^M\left({L}_{ij k}\right)$, which is computed as follows:

4 $$ {l}_{ij}=\left(-2\right)\frac{1}{M}{\sum}_{k=1}^M\log \left({\prod}_{g= AA, AB, BB}\left({f}_{jk(x)}^{1_{x=g}}\right)\right) $$

For simplicity, the above is denoted by -2logLikehood hereafter. To avoid calculating the logarithm on zero counts of genotypes, each genotype frequency was re-computed based on allele frequencies estimated based on a Bayesian Binomial model. Assume a conjugate Beta prior for *q*, that is, *p*(*q*) = *Beta*(*α*, *β*), where *q* is the frequency of say allele B, and *α* and *β* are hyper-parameters in the prior distribution, the posterior distribution of *q* is also a Beta distribution function:

5 $$ \left.q\right|x,N\sim Beta\left(2{n}_{BB}+{n}_{AB}+a,2{n}_{AA}+{n}_{AB}+\beta \right) $$

where *N* = 2(*n*_*AA*_ + *n*_*AB*_ + *n*_*BB*_). Denote $\widehat{q}$ to be the posterior mean of *q*. Then, assuming Hardy-Weinberg equilibrium, the frequencies of genotypes AA, AB, and BB, respectively, were given as follows:

6 $$ {\displaystyle \begin{array}{c}{f}_{AA}={\left(1-\widehat{q}\right)}^2={\left(1-\frac{2{n}_{BB}+{n}_{AB}+a}{2\left({n}_{AA}+{n}_{AB}+{n}_{BB}\right)+a+\beta}\right)}^2\\ {}={\left(\frac{2{n}_{AA}+{n}_{AB}+\beta }{2\left({n}_{AA}+{n}_{AB}+{n}_{BB}\right)+a+\beta}\right)}^2\end{array}} $$

7 $$ {\displaystyle \begin{array}{c}{f}_{AB}=2\times \widehat{q}\left(1-\widehat{q}\right)\\ {}=2\times \frac{2{n}_{BB}+{n}_{AB}+a}{2\left({n}_{AA}+{n}_{AB}+{n}_{BB}\right)+a+\beta}\times \left(1-\frac{2{n}_{BB}+{n}_{AB}+a}{2\left({n}_{AA}+{n}_{AB}+{n}_{BB}\right)+a+\beta}\right)\\ {}=2\times \frac{2{n}_{BB}+{n}_{AB}+a}{2\left({n}_{AA}+{n}_{AB}+{n}_{BB}\right)+a+\beta}\times \left(\frac{2{n}_{AA}+{n}_{AB}+\beta }{2\left({n}_{AA}+{n}_{AB}+{n}_{BB}\right)+a+\beta}\right)\end{array}} $$

8 $$ {f}_{BB}={\widehat{q}}^2={\left(\frac{2{n}_{BB}+{n}_{AB}+a}{2\left({n}_{AA}+{n}_{AB}+{n}_{BB}\right)+a+\beta}\right)}^2 $$

Each reference animal had a value of *l*_*ij*_ which was smaller than a pre-defined cutoff, *l*_*α*_, where, for example, *l*_*α* = 0.99_ represented the 99% quantile of *l*_*ij*_ values. After removing outliers for each breed (described later), allele frequencies of the reference SNP panels were re-computed using reference animals only and the updated allele frequencies of reference SNPs were used in the estimation of GBC. Note that the above were illustrated using the AB genotype notation. The same principles apply to the ACGT genotype notation as well.

### Estimation of genomic breed composition

#### Linear regression model

The linear regression approach estimated GBC for each animal by regressing discrete random variables (genotypes of this animal) corresponding to counts of certain alleles of reference SNPs across the genome on the corresponding allele frequencies of each reference SNP in a number of reference populations [15, 17]. Let **y** be an *M* × 1 vector of genotypes for each animal, where *M* is the number of reference SNPs, and genotypes were coded as the number of B alleles of each reference SNP observed on each animal. Let ***F*** = {*f*_*kj*_} be an *M*×*T* matrix, where *f*_*kj*_ was the frequency of B allele of SNP *k* pertaining to population *j*, and *T* is the number of breeds. Then, GBC was estimated based on the following linear model:

9 $$ \boldsymbol{y}=\mathbf{1}\boldsymbol{\mu } +\boldsymbol{Fb}+\boldsymbol{e} $$

where *μ* is the overall mean, and ***b*** is a *T* × 1 vector of regression coefficients, each pertaining to a breed, and ***e*** is a residual term. Note that the sum of regression coefficients across the *T* breeds computed for each animal did not equal to 1, and adjustment of these regression coefficients were needed to restrict the sum of regression coefficients per animal to be 1. VanRaden and Cooper (2015) proposed a method to adjust breed regression coefficients [12], but their method is not straightforward to follow. In this study, we proposed an approximate approach, which was simple yet effective, as follows. For each animal, all negative regression coefficients, if any, were replaced by zeros. Then, for each animal, the GBC of a breed was estimated to be the ratio of that breed regression coefficient over the sum of the regression coefficients across all *m* breeds.

#### Admixture model

Given allele frequencies for a number of SNPs which had been estimated for each reference breed, an individual’s genotypes at these loci was modeled as an admixture of multiple breeds [18]. The admixture coefficients of the *T* breeds, computed for each animal, corresponded to the fractions of the individual’s genome which was derived from each reference breed, and they provided estimates of GBC of the *T* breeds for each animal. In the admixture model, the value of each admixture coefficient was between 0 and 1, and the sum of admixture coefficients (GBC) computed for each animal is always 1 under the assumption of 100% genetic contributions by the *T* known breeds to this individual animal. For a given animal, if the admixture coefficient of a single reference breed was 1 (or close to 1), then this animal was identified as a purebred animal of that breed.

Consider an individual, say *i*, with observed genotype for a SNP, say *k*. Let A and B be the two alleles of this SNP. There were three possible genotypes: AA, AB and BB, respectively. Assuming Hardy-Weinberg equilibrium, the probability of observing each genotype on this animal were given as follows:

10 $$ \mathit{\Pr}\left({g}_{ik}|{q}_{ik}\right)=\left\{\begin{array}{c}{\left(1-{q}_{ik}\right)}^2\kern6.5em {g}_{i\mathrm{k}}\kern0.5em =0\ (AA)\\ {}2{q}_{ik}\left(1-{q}_{ik}\right)\kern4.75em {g}_{ik}=1\ (AB)\\ {}\ {q}_{ik}^2\kern9.75em {g}_{ik}\kern0.5em =2\ (BB)\end{array}\right. $$

In the above, *q*_*ik*_ was the weighted frequency of allele *B* of the *k*-th SNP, pertaining to the admixture of the *i*-th individual, and its quantity was given by ${q}_{ik}={\sum}_{j=1}^T{w}_{ij}{f}_{jk}$, where *w*_*ij*_ was an weight of the *j*-th breed contributing to the admixture of the *i*-th individual, and *f*_*jk*_ was the allele *B* frequency of the *k*-th SNP in the *j*-th reference breed.

Denote ${\boldsymbol{w}}_{\boldsymbol{i}}=\left({w}_{i1}\kern0.5em {w}_{i2}\kern0.5em \dots \kern0.5em {w}_{iT}\right)$ to be a vector of the weights of *T* breeds, and $$ {\boldsymbol{g}}_i=\left({g}_{i1}\kern0.5em {g}_{i2}\kern0.5em \begin{array}{cc}\dots \kern0.5em {g}_{iM}\end{array}\right) $$ be a vector of observed genotypes of the *M* reference SNPs, both pertaining to individual *i*. Then, the log-likelihood pertaining to this individual was given by the following:

11 $$ {\displaystyle \begin{array}{c}L\left({\boldsymbol{w}}_{\boldsymbol{i}}\right)={\sum}_{k=1}^M\mathit{\ln}\left(\mathit{\Pr}\left(\left.{g}_{ik}\right|{q}_{ik}\right)\right)\\ {}=\left[{\sum}_{k=1}^M{g}_{ik}\mathit{\ln}\left({q}_{ik}\right)+\left(2-{g}_{ik}\right)\mathit{\ln}\left(1-{q}_{ik}\right)\right]+C\end{array}} $$

where *C* was a constant. Note that the above assumes that all SNPs were independent or in linkage equilibrium with each other, which might not hold for high-density SNPs. But this assumption was taken to be approximate for low density SNP panels. A practical solution to accommodate this assumption would be to prune SNPs to reduce the linkage disequilibrium (LD) between the markers [16]. Given SNP allele frequencies for the *T* reference breeds and genotypes of these SNPs for a test animal, say *i*, the solutions of breed admixture coefficients for this animal is obtained by maximizing *L*(***w***_***i***_), under the constraints *w*_*ij*_ ≥ 0 and ${\sum}_{j=1}^T{w}_{ij}=1$.

A variety of optimization methods are available for estimating the above admixture coefficients. Newton’s method involves the manipulation and inversion of a possibly large matrix, which can be computationally intensive [24]. The EM algorithm [28] has been implemented in some relevant software packages, such as FRAPPE [23], but this algorithm has slow convergence. We used the Broyden-Fletcher-Goldfarb-Shanno (BFGS) method [18] to optimize likelihood function (11). The BFGS algorithm is a popular quasi-Newton method for solving non-linear optimization problems, which utilizes the first derivatives of the likelihood function and approximates the Hessian matrix of the second derivatives [29].

Computationally, an iterative approach was used to find a parsimonious set of GBC values for an individual by iteratively removing breed(s) for which a nonzero admixture coefficient does not improve the model fitting significantly [18]. This procedure was analogous to backwards elimination variable selection using the likelihood ratio statistic. Briefly, this approach proceeded as follows: (1) Calculate the maximum likelihood estimate for the vector of admixture coefficients (***w***_***i***_); (2) For each breed, say *j,* with a non-zero admixture coefficient, calculate *δ*_*ij*_ = *L*_*max*_ − *L*_−*j*_ obtained by calculating the maximum likelihood fit with the *j*-th admixture coefficient constrained to be 0; (3) Determine the breed with the smallest value of *δ*_*ij*_*;* (4) Set for admixture coefficient *w*_*ij*_ to be 0 if *δ*_*ij*_*&lt; τ,* where *τ* was a threshold based on the likelihood ratio test; Repeated Steps 2–4 until the changes in the likelihood was acceptably minimized.

#### Impact on genomic prediction

GBC were computed for 1424 cattle putatively presented as Santa Gertrudis. These animals were not included in the reference set to define the Santa Gertrudis breed allele frequencies. Based on the density plot of calculated GBC of Santa Gertrudis for these animals (Additional file 1: Figure S1), all the animals were assigned into three groups with varying GBCSG levels: 0 ≤ GBCSG &lt; 0.70 (71 cattle), 0.70 ≤ GBCSG &lt; 0.90 (128 cattle), and GBCSG ≥0.90 (1,225 cattle). Animals in the last group was considered to be purebred.

The phenotypes included expected progeny differences (EPD) of birth weight (BW), fat thickness (FAT), hot carcass weight (HCW), marbling score (MARB), ribeye area (REA), scrotal Circumference (SC), weaning weight (WW), maternal weaning weight (MWW), and yearling weight (YW). Summary statistics of EPDs and accuracies of EPDs of the nine traits are shown in Table 2. The mean accuracies of EPD for the nine traits were generally low, ranging from 0.039 (SC) to 0.297 (WW). The maximum accuracy of EPDs for the nine traits were between 0.599 (SC) and 0.887 (WW). These EPDs were de-regressed following Garrick et al. (2009) [30]. After data cleaning, molecular EBV (MEBV) was computed to be the sum of the effects of 37,775 SNPs that each individual animal carried. Then, GPA on the nine traits were measured as correlations between deregressed EBV (dEBV) and MEBV. In the 1,225 Santa Gertrudis cattle (GBCSG &gt; 0.90), GPA were evaluated by leave-one-out cross-validation (LOOCV). Briefly, in the 1,225 animals with GBCSG ≥0.90, SNP effects were estimated on a set of 1,224 randomly selected animals (i.e., training set) and then tested on the remaining individual (i.e. test set). This procedure rotated 1,225 times such that each individual was used in the test set once and only once. In the two groups with lower GBCSG (0 ≤ GBCSG &lt; 0.70; 0.75 ≤ GBCSG &lt; 0.90), GPA were evaluated with their MEBV computed by SNP effects estimated previously from the 1,225 Santa Gertrudis cattle with GBCSG ≥0.90. In a broader sense, this is similar to assessing the predictability of SNP effects obtained from pure-bred animals on their crosses or animals mixed from other breeds, though Santa Gertrudis is itself an established composite cattle breed.

**Table 2:** Summary statistics of expected progeny differences (EPD) and accuracies of EPD of nine quantitative traits for 1424 animals presented as Santa Gertrudis

Trait	N	Min	Q25%	Median	Q75%	Max	Mean	SD
EPD								
BW, lb	1424	−8.411	−0.725	−0.263	0.381	6.742	−0.14	0.992
FAT, in	1424	−0.125	−0.002	0.001	0.002	0.062	0	0.006
HCW, lb	1424	− 35.79	− 4.188	− 1.01	3.795	39.95	0.145	7.041
MARB^a^	1424	−0.329	− 0.017	0.004	0.012	0.473	−0.004	0.04
MWW, lb	1424	−23.04	−2.051	0.4555	2.783	19.63	0.311	4.393
REA, sq. in	1424	−0.551	−0.041	0.003	0.043	0.647	0.005	0.091
SC, cm	1424	−1.044	−0.069	0.017	0.072	1.24	−0.008	0.152
WW, lb	1424	−32.37	−4.211	−1.118	4.175	46.97	0.235	7.093
YW, lb	1424	−45.82	−5.263	−1	5.62	54.39	0.702	9.901
Accuracy of EPD								
BW	1424	0.001	0.049	0.136	0.174	0.852	0.279	0.149
FAT	1424	0.001	0.006	0.023	0.081	0.714	0.097	0.125
HCW	1424	0.001	0.022	0.066	0.099	0.574	0.139	0.099
MARB	1424	0.001	0.003	0.014	0.059	0.628	0.069	0.095
MWW	1424	0.001	0.073	0.161	0.181	0.84	0.269	0.128
REA	1424	0.001	0.01	0.032	0.069	0.595	0.089	0.088
SC	1424	0.001	0.003	0.009	0.051	0.599	0.039	0.097
WW	1424	0.001	0.059	0.158	0.191	0.887	0.297	0.151
YW	1424	0.001	0.034	0.098	0.135	0.714	0.197	0.126

*Min* minimum value, *Median* median value (50% quantile), *Max* maximum value, *QX%* X% quantile, where X = 25 and 75, respectively, *SD* standard deviation, *BW* birth weight, *WW* weaning weight, *HCW* hot carcass weight, *MARB* marbling score, *MWW* maternal weaning weight, *FAT* fat thickness, *REA* ribeye area, *SC* scrotal circumference, *YW* yearling weight

^a^4.00 = Slight, 5.00 = Small, 6.00 = Modest, 7.00 = Moderate, 8.00 = Slightly Abundant

## Results

### Reference SNPs

**Fig. 1:**
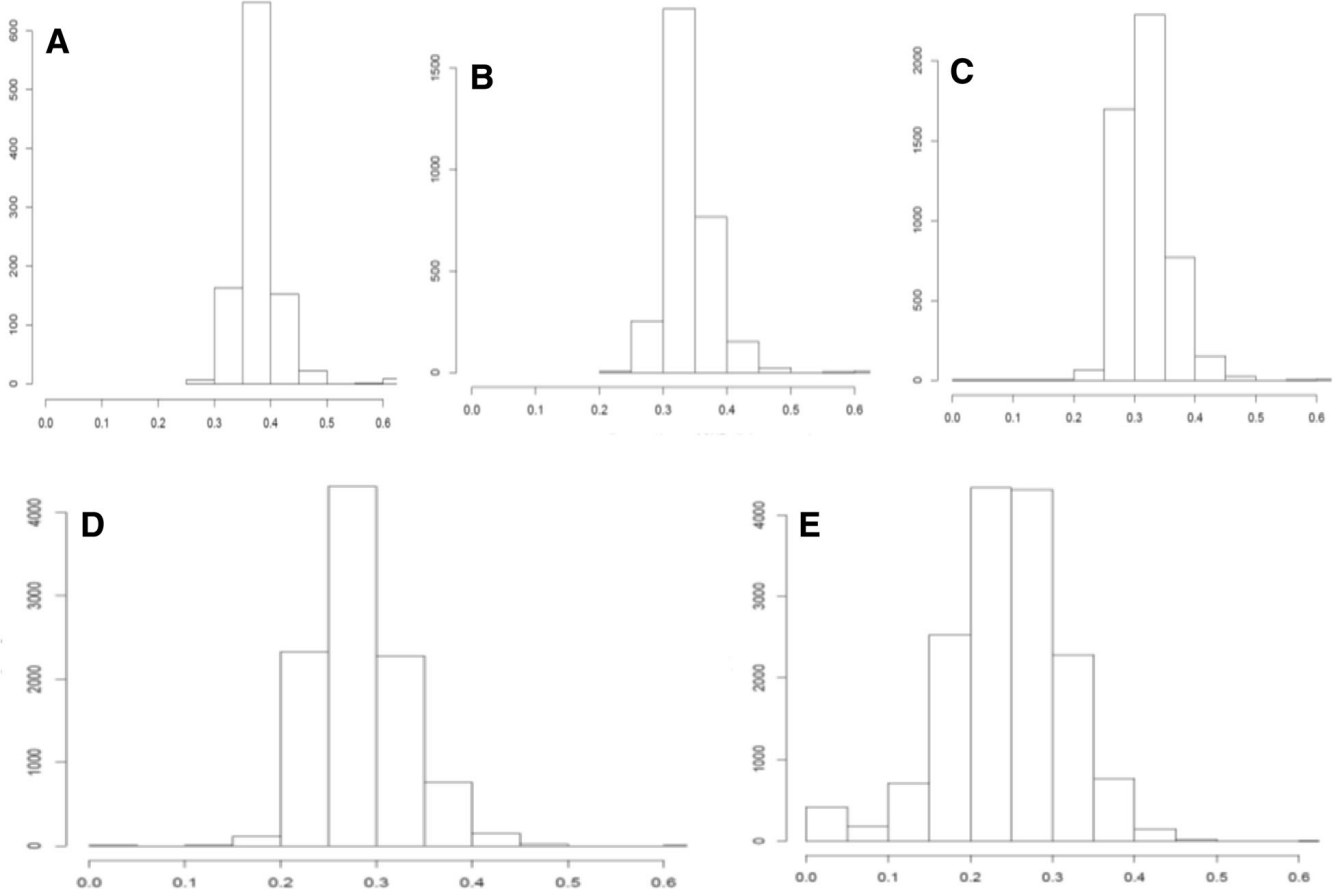
Average Euclidean distance of SNP allele frequencies for each of the five SNP panels. **a** = 1,000 SNPs; **b** = 3,000 SNPs; **c** = 5,000 SNPs; **d** = 10,000 SNPs; **e** = 15,708 SNPs. The X-axis represents average Euclidean distance (AED) between SNP allele frequencies, and the y-axis represents frequencies of SNPs for a given level of AED

Average Euclidean distance among the ten breeds computed by each of the five SNP panels increased as the panel size decreased. The AED of reference SNP allele frequencies were 0.243 (16 K), 0.285 (10 K), 0.319 (5 K), 0.340 (3 K), and 0.377 (1 K), respectively. This trend indicates that maximizing AED have successfully led to the inclusion of highly informative SNPs in each of the four selected panels (Fig. 1a-d). For example, a dominating majority of SNPs in the 1 K panel had AED values greater than 0.3. In contrast, the unselected 16 K panel had a considerable number of low-informative SNPs with close to zero AED among the ten breeds, and a majority of SNPs in the 16 K panel had AED less than 0.3 (Fig. 1e).

### Reference animals

The likelihood values of an animal belonging to the reference breed, computed using each of the five SNP panels respectively, were highly correlated among the five SNP panels. In the 198 animals putatively presented as Akaushi, Pearson correlations of -2logLikelihood values computed by these five SNP panels varied between 0.994 and 0.999 and the corresponding Spearman rank correlations ranged from 0.858 to 0.991 (Additional file 7: Table S1). For example, plots of -2logLikelihood values obtained from either 1 K or 16 K SNP panels are shown in Additional file 3: Figure S3 with high concordance of computed -2logLikelihood values between the two panels.

**Fig. 2:**
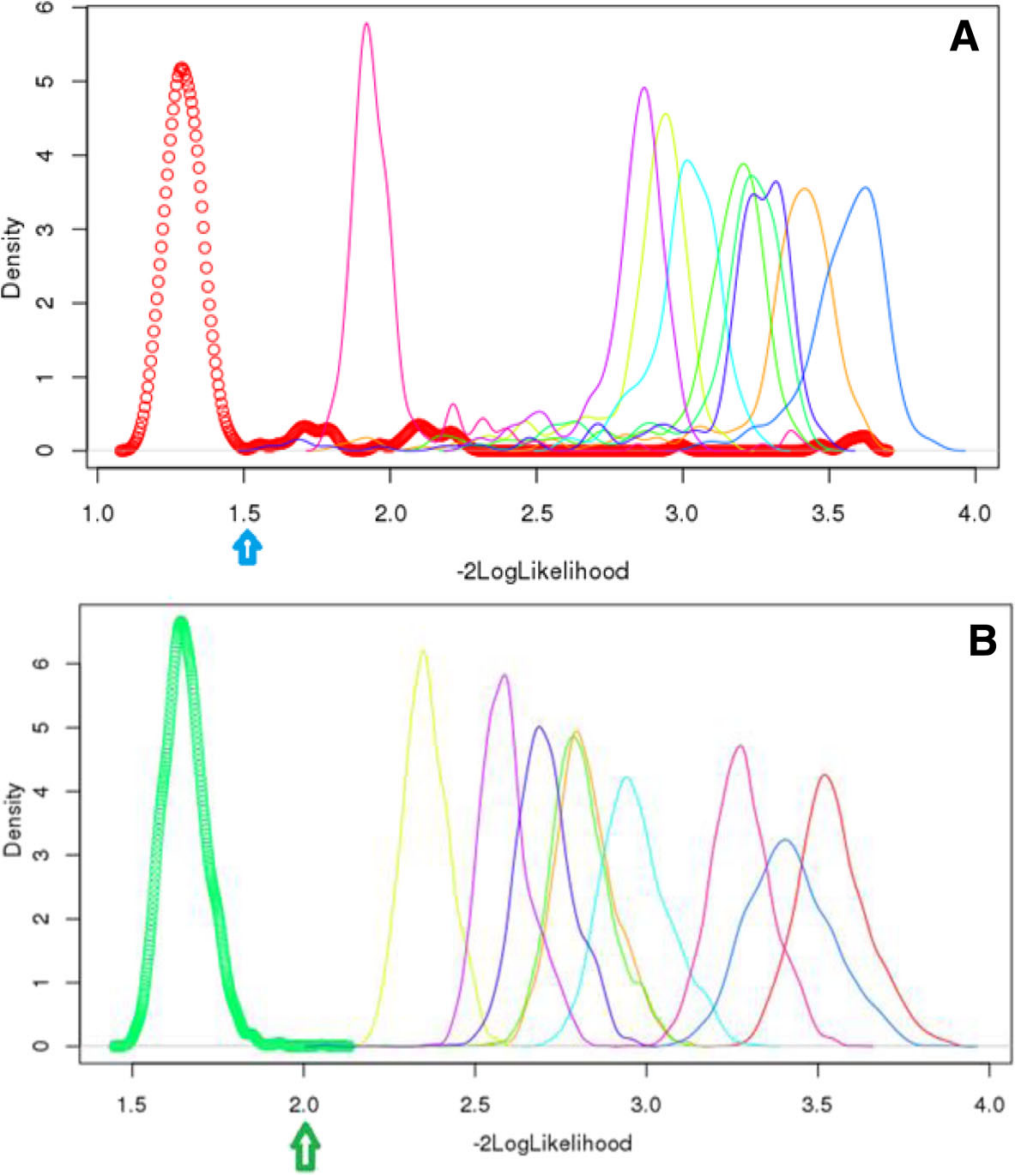
Plots of -2logLikelihood for: (**a**) 198 animals presented as Akaushi (red circles) and (**b**) 2,423 animals presented as Hereford (green circles), obtained with assumed true allele frequencies of SNPs for each of the 10 breeds. The blue arrow below the x-axis indicates the cutoff value of the -2logLikelihood values for data cleaning. Assumed breeds from left to right are: (**a**) Akaushi, Wagyu, Santa Gertrudis, Beef Master, Holstein, Brangus, Hereford, Red Angus, Angus, and Jersey; (**b**) Hereford, Beef Master, Santa Gertrudis, Red Angus, Brangus, Angus, Holstein, Wagyu, Jersey, and Akaushi

The 1 K panel was used to select reference animals for each breed. Animals with -2logLikelihood exceeding a given cutoff value were excluded from the reference animals for each breed. These cutoff values differed by breeds, which were taken based on visual evaluation of the distribution of -2logLikelihood values of all the animals for each breed. For examples, plots of the distributions of -2logLikelihood obtained using the 1 K SNP panel for the 198 animals presented as Akaushi and the 2,423 animals presented as Hereford showed the presence of outliers (Fig. 2a and b, respectively). Based on these two graphs, the cutoff value of -2logLikelihood of 1.5 was used for Akaushi cattle and 2.0 was used for Hereford cattle, which represented a cutoff at 84.34% quantile for Akaushi cattle and a cutoff at 99.53% quantile for Hereford cattle. The number of reference animals for each breed, after removing outliers, are listed parenthetically in Table 1. After finalizing the reference animal sets, allelic and genotype frequencies of reference SNPs were re-computed based on selected reference animals.

**Fig. 3:**
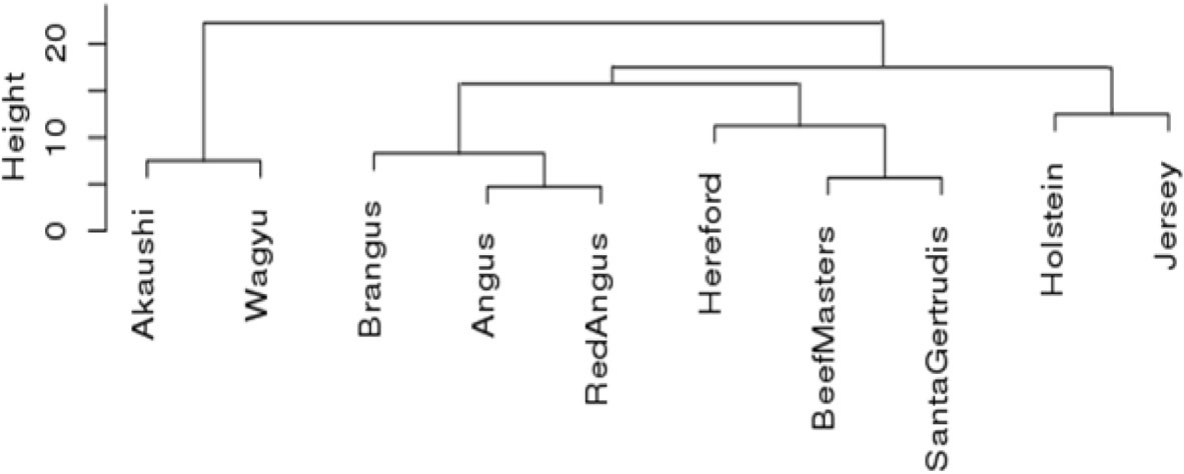
Hierarchical clustering of 10 cattle breeds based on Euclidean distance of allele frequencies of SNPs on the 1 K SNP panel

Hierarchical cluster analysis based on Euclidean distances of the 1 K SNP allele frequencies among the ten breeds assigned these ten bovine breeds into four groups (Fig. 3). The first was the Japanese cattle group, which included Akaushi and Wagyu. Then, there were two more beef cattle groups, one consisted of Angus, Red Angus, and Brangus; the other consisted of Beefmaster, Santa Gertrudis, and Hereford. These last two beef groups were distantly related possibly because they shared common remote ancestries. For example, the Brangus breed was developed to utilize the superior traits of Angus and Brahman cattle, and their registration standard was stabilized at pedigree estimated 3/8 Brahman and 5/8 Angus [31], whereas Santa Gertrudis cattle were also the descendants of 3/8 Brahman cattle and 5/8 Shorthorn [27]. The fourth major group was the dairy cattle group, which included Holstein and Jersey, with their relationship being the most distant of the four groups. The dairy groups were more related with western beef cattle and American beef composites than Japanese beef cattle.

### Estimated GBC for Akaushi cattle

#### Admixture model

Genomic breed composition was estimated for the 198 animals putatively presented as Akaushi using the five SNP panels based on the admixture model and the linear regression model, respectively (Table 3). The results obtained using the admixture model agreed very well among the five SNP panels, which identified 166 animals with GBC of Akaushi being equal to 1. Hence, these animals were considered to be purebred Akaushi cattle. There were 27 animals with GBCA &lt; 1 based on each of the five panels. Arguably, animal X167 might still be a purebred Akaushi cattle (which actually is) because its GBCA ≈ 0.93. Hence, the mis-classification rate was 0.60% (=1/167) if using GBCA = 1 as the cutoff. The remaining animals were either crossbreds between Akaushi cattle or animals from other beef breeds. It came to our attentions that there were five animals which had 0% GBCA, which suggested that they had no Akaushi inheritance. In fact, these five animals were mixed Red Angus cattle.

**Table 3:** Distribution of genomic breed composition (GBC) of 198 animals presented as Akaushi

Akaushi breed coefficient	Admixture model					Linear Regression model				
	1 K	3 K	5 K	10 K	16 K	1 K	3 K	5 K	10 K	16 K
=1	166	166	166	166	166	57	125	142	150	151
[0.9, 1.0)	1	1	1	1	0	71	36	24	18	17
[0.8, 0.9)	9	8	9	9	11	41	13	9	7	8
[0.7, 0.8)	4	5	4	4	3	8	4	5	5	4
[0.6, 0.7)	1	0	1	1	1	3	4	1	1	3
[0.5, 0.6)	11	12	11	11	11	6	9	11	11	9
[0.4, 0.5)	0	0	0	0	0	5	1	0	0	0
[0.3, 0.4)	0	0	0	0	0	1	0	0	0	0
[0.2, 0.3)	0	0	0	0	0	0	0	0	0	0
[0.1, 0.2)	1	1	1	1	1	1	1	1	1	1
[0, 0.1)	5	5	5	5	5	5	5	5	5	5

[x,y) = an interval of GBC in which the value is greater than (or equal to) x and less than y

For the 198 animals, -2logLikelihood values were computed using the 1 K panel and 16 K panel, respectively, which correlated very well with each other (Additional file 3: Figure S3). Based the 1 K panel, for example, the 166 animals with -2logLikelihood &lt; 1.440 were all assigned to be 100% purebred Akaushi cattle (GBCA = 1), whereas, the remaining crossbreds and non Akaushi animals had -2logLikelihood &gt; 1.440 (Additional file 7: Table S2). In general, the larger value of -2logLikelihood that an animal had, the less likely for it to be a purebred animal. The results obtained from the likelihood-based approach agreed well with estimated GBC of individual animals based on the admixture model.

### Linear regression

**Fig. 4:**
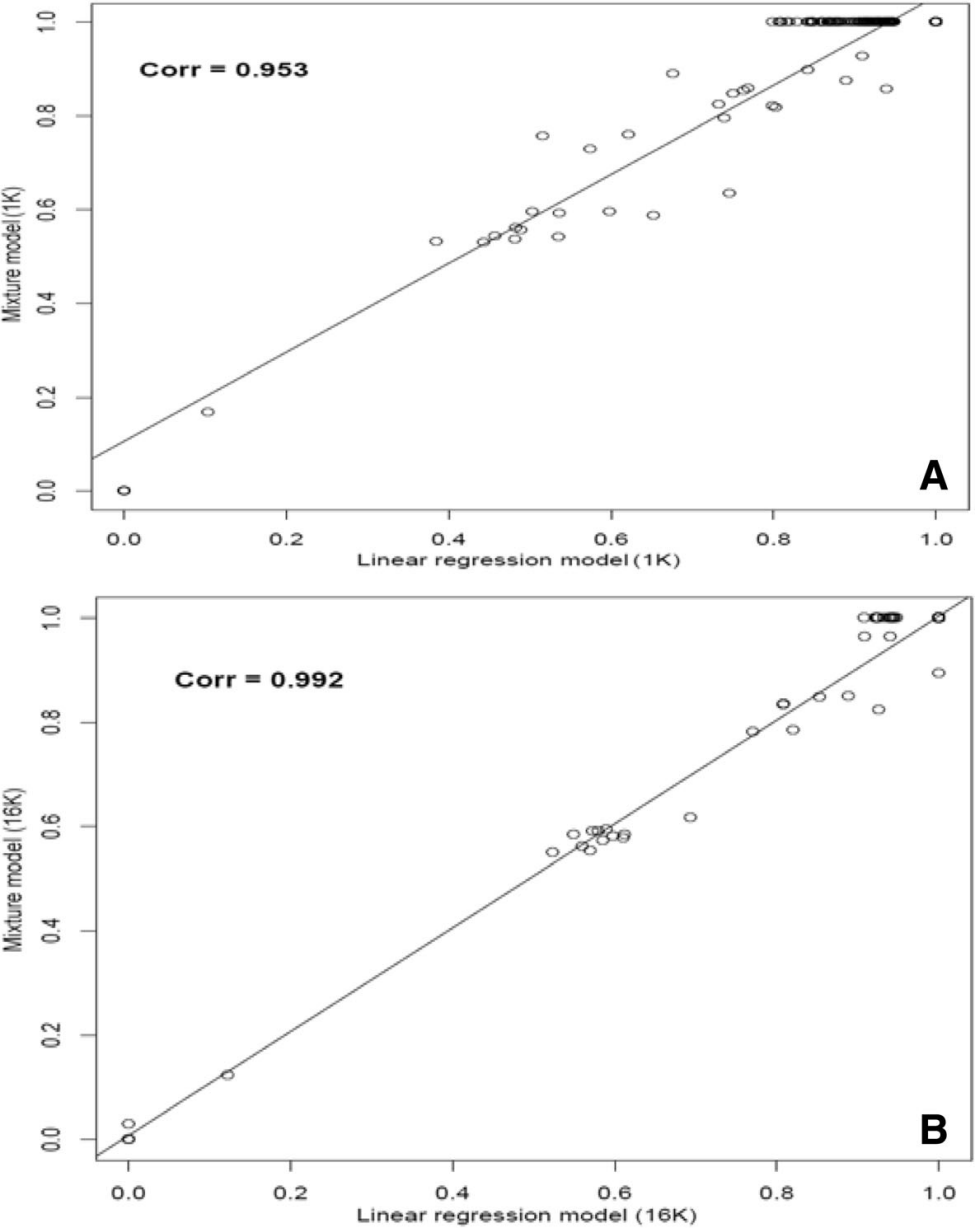
Plots of genomic breed composition (GBC) of 198 animals presented as Akaushi based on an admixture model versus a linear regression model: (**a**) GBC were estimated using 1 K SNP panel; (**b**) GBC were estimated using 16 K SNP panel

The results from the linear regression method, however, showed considerable differences among the five panels. The numbers of animals with GBCA = 1 increased with the number of SNPs in the panel (Table 3). If using GBCA = 1 as the cutoff, the number of purebred animals identified by these panels were 57 (1 K), 125 (3 K), 142 (5 K), 150 (10 K) and 151 (16 K), respectively. Apparently, the linear regression model reported less animals with GBCA = 1 than the admixture model, and the regression approach seemingly required the use of more SNP in order to give comparable results to the admixture model. Roughly speaking, animals with GBCA = 1 identified by the admixture model corresponded to those with GBCA &gt; 0.9 (5 K to 16 K) or GBCA &gt; 0.8 (1 K and 3 K) based on the linear regression model. Nevertheless, plots of GBCA obtained using the admixture model versus those obtained using the linear regression model showed high correlations (r = 0.953 to 0.992) based on 1 K SNP panel and 16 K SNP panel, respectively (Fig. 4).

### Genomic prediction in Santa Gertrudis cattle

**Fig. 5:**
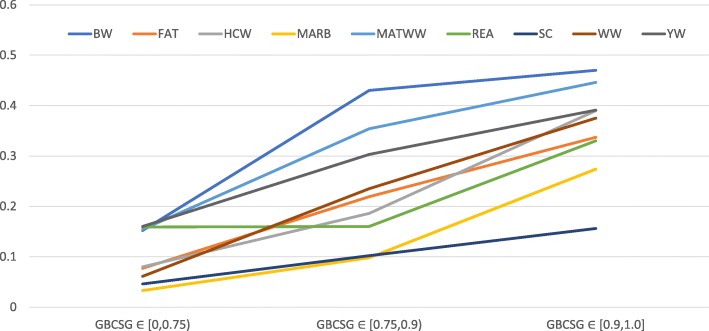
Plots of genomic prediction accuracies on nine traits in 1424 beef cattle with varying level of genomic breed composition of the Santa Gertrudis breed (GBCSG). BW = birth weight; WW = weaning weight; HCW = hot carcass weight; MARB = marbling score; MWW = maternal weaning weight; FAT = fat thickness; REA = ribeye area; SC = Scrotal Circumference; YW = yearling weight; IVA = independent validation on 71 cattle with 0 ≤ GBCSG &lt; 0.70 while SNP effects were estimated from the 1225 animals with GBCSG ≥0.90; IVB independent validation on 128 cattle with 0.70 ≤ GBCSG &lt; 0.90 while SNP effects estimated from the 1225 animals with GBCSG &gt; 0.90; LOOCV = leave-out cross-validation in the 1225 animals with GBCSG ≥0.90

SNP effects were estimated and validated by leave-one out cross-validation in 1,225 animals with GBCSG equaled to or greater than 0.90. Predictability of these SNP effects were also tested in the remaining animals with GBCSG less than 0.90. The latter were considered to be cross-bred of Santa Gertrudis cattle, which included 25 animals with GBCSG &lt; 0.5. Genomic prediction accuracies on the nine traits ranged from 0.156 (SC) to 0.470 (BW) in the 1,225 GBCSG-validated Santa Gertrudis cattle (GBCSG ≥0.90). Prediction accuracies on the nine traits using these SNP effects, however, decreased in the other two groups as GBCSG became smaller, which were between 0.102 (SC) and 0.430 (BW) when 0.70 ≤ GBCSG &lt; 0.90, and between 0.033 (MARB) and 0.160 (YW) when 0 ≤ GBCSG &lt; 0.70 (Fig. 5).

## Discussion

### Selection of reference SNPs and reference animals

Estimation of GBC was evaluated using two statistical models: admixture model and regression approach. Selection informative SNPs is not a necessary step for the regression approach. Often, using high-density SNPs in the regression approach tends to give more reliable results, though the computing may take more time. However, an admixture model directly using high-density SNPs is highly computational demanding and inefficient. Thus, it is very necessary to select low-density panels for estimating GBC. The present results indicated that SNP panels for estimating GBC were effectively obtained by maximizing AED among breeds, which successfully included highly informative SNPs in each of the four selected panels (Fig. 1a-d). Map view of the five SNP panels showed that these selected SNPs were located on all the 29 autosomes and X chromosome (SNPs on Y chromosome were not included as candidate SNPs), but their distributions on each chromosome were very uneven, in particular, for the 1K – 5K panels (Additional file 2: Figure S2). This result might reflect the consequences of selection dynamics, either natural or artificial, and that of genetic drift on the differentiation of these breeds during the history of adaption and development of these breeds, during which their genomes may have been shuffled considerably.

Selecting (or validation) of reference animals is a necessary step because these reference animals are supposed to be representative of their respective breeds, and including cross-bred animals or even animals from other breeds would mis-lead the conclusion. We employed a likelihood-based approach to select reference animals for each breed. Alternatively, outliers can be identified using a standard deviation approach or Tukey’s method. The latter was the statistical method underlying the boxplot in R, which uses interquartile (IQR) range approach, and outliers are identified as ranges above or below the 1.5IQR. Validation of these animals by their pedigrees and breed registry records is also important when the latter information is available. In the present study, animals with -2logLikelihood less than the cutoff value were retained as reference animals for each breed, because they are more likely to be a purebred animal (or an animal which is representative of each breed). Note that choosing the cutoff value for each breed is subject to the presence of outliers. Keeping outliers leads to high error rates of false positives (i.e., an animal is identified as a member of a given breed but it is not). On the other hand, if the exclusion rate is too stringent (meaning that more animals than crossbreds are removed), it would bias SNP allele frequencies of a given population toward another extreme, which in turn results in higher errors of false negatives (i.e., an animal is claimed to be not belonging to that population but it is). Ideally, with good quality data, the percent of eliminated animals is expected to be less than 5%. In the present studies, the percent of eliminated animals was generally below 5%, ranging from 0.45% for Hereford to 15.66% for Akaushi. Of the 8,905 registered Holstein animals, for example, 96.4% of these animals had GBC of Holstein (GBCH) equal to 1, 98.7% had GBCH ≥0.90, and 99.6% had GBCH ≥0.80. Based the distribution of their GBCH values, approximately 0.47% of these animals were identified as outliers and excluded from the reference group. For the three composite cattle breeds (Brangus, Beef Master and Santa Gertrudis), though two founder breeders (Brahman and Shorthorn) were not present, the three composite breeds could be distinguished from each other because they had unique patterns of allele frequencies (and thus their breed likelihood values) (Additional file 4: Figure S4; Additional file 5: Figure S5; Additional file 6: Figure S6).

Five SNP panels were selected and used in the present study, which varied from 1K to 16K. Frkonja et al. (2012), who also used the admixture model, found that a relatively small number of SNPs (approximately 4000 randomly selected) would suffice to predict breed composition [32]. Nevertheless, our results suggested that the number of SNPs could be much smaller if these SNPs were optimally selected (data not presented). Potentially, the number of SNPs can be further reduced if SNPs are pruned to ensure reduced LD. Though searching for minimum SNP panel sizes for estimation GBC is of interest, this topic was not investigated in the present study.

### Admixture model versus regression approach

Genomic breed composition was estimated for the 198 animals putatively presented as Akaushi using the five SNP panels based on the admixture model and the linear regression model, respectively. The two models had varied to some extent concerning the number of animals with GBCA = 1. Estimated breed compositions for the 198 animals based on the admixture model agreed very well among the five SNP panels, which consistently identified 166 animals with GBC of Akaushi being equal to 1. However, the results from the linear regression method showed considerable differences among the five panels, and the numbers of animals with GBCA = 1 increased with the number of SNPs included in the regression model. Hence, we expect that the regression model will give more reliable results using high-density SNPs. Given low-density SNP panels, the admixture model yielded more consistent results among the five selected panels than the linear regression model.

Nevertheless, the admixture model is more computational intensive, and it had stronger assumptions. Specifically, the admixture model assumes that SNP loci are independent of each other. This assumption typically does not hold with high-density SNPs. A practical way of reducing LD is to prune SNPs. In the present study, however, we did not prune SNPs but instead used all selected low-density SNPs which are highly informative of these population relationships. Use of low-density SNPs tended to reduce LD considerably. By computing GBC locus-wise and assuming complete independence of these the involving SNPs, this type of locus-wise genomic breed composition is well explained by genomic similarity due to identical by state (IBS), rather than being identical by descent (IBD). In this sense, computed GBC can be more precisely described by genomic breed similarity (GBS). Alternatively, GBC can be computed considering only alleles located within runs of homozygosity (ROH), which represents IBD more than IBS because the probability for a large segment of chromosome to be IBD is high. Arguably, this allowed the use of SNPs in high LD and computed GBC could be better captured via the genomic similarity IBD than a random set of evenly-spaced SNPs, because it tended to give more weight to SNPs on ROH.

### Impact on “down-stream” genomic prediction accuracy

Generally speaking, genomic prediction accuracies on the nine traits were low, possibly because the accuracies of EPD were low, and the de-regressed EBV could have more noise. Our results showed that SNP effects obtained from 1,225 animals with GBCSG equaled to or greater than 0.90 were more predictable in this same set of animals per se, as evaluated by leave-one validation, than the other two sets of animals which were considered to be crossbreds of Santa Gertrudis animals (Additional file 7: Table S3). Also possibly, animals with very low GBCSG might not truly be “crosses” of Santa Gertrudis, but they could be individual animals in that breed whose genotypes suggested significant deviations from the patterns of allele frequencies of that breed, due to genetic sampling or segregation in the progeny. Nevertheless, these results suggest that animals differed in estimated GBC also varied in their genetic architecture of quantitative traits. In a broader sense, estimated SNP effects in certain breed does not necessarily apply well to animals of a different breed, and genomic predictions built for purebred animals do not necessarily work well on low percentage crosses with that breed. Thus, knowing GBC of individual animals helps characterize predictability of genomic potential of animals more precisely.

Genomic prediction of crossbred animals is of interest. This usually requires that a sufficient number of crossbred animals with genotypes and phenotypes be included in the training set, which however is often difficult to obtain. Instead, there were evidences that genomic prediction on crossbred animals could be improved by taking their GEBVs to be weighted averages of direct genomic values computed from SNP effects for each of the pure breeds and the weights were each animal’s GBC [12]. This is an application of practical interest, which remains to be further investigated in future studies.

## Conclusions

Five SNP panels (1 K, 3 K, 5 K, 10 K, and 16 K) were designed for estimating genomic breed composition in cattle. The 16 K panel consisted of common, informative SNPs on five currently available commercial bovine SNP chips. From the 16 K SNP panel, four smaller SNP panels (1 K, 3 K, 5 K, and 10 K) were optimally selected by maximizing AED of allelic frequencies of SNPs among ten cattle breeds. The availability of these selected SNP panels facilitates breed identification and estimation using currently available commercial bovine SNP chips without the need to design new SNP chips or pay extra lab genotyping cost. These results from the admixture model showed that the five SNP panels performed very similarly in the estimation of GBC in 198 animals putatively presented as Akaushi. Overall, our results are highly comparable to admixture models, e.g., the one proposed by Bansal-Libiger (2015) and implemented by the iAdmix program, because we share the same statistical framework. The admixture model differed from the linear regression approach in number of animals with purebred coefficient being exactly equal to 1, but estimated GBC from both methods were highly correlated (&gt; 90%). Yet, our results did not suggest that the two methods contradicted with each other, but that the linear regression approach need to have more reference SNPs than the admixture model to give comparable results.

In view of utility, the 1 K panel is the most cost effective among the five SNP panels for estimating GBC but the two larger SNP panels (10 K and 16 K) can be more robust as an independent LD SNP panel if imputation to moderate- or high-density SNP genotypes is a necessary task. The present study did not search for a minimum number of SNPs for estimating GBC. This was an interesting topic but it was not of direct relevance in the present study. In the present study, 1 K to 5 K SNPs are desirable sizes for reliably estimating GBC and they are convenient to be included as core content for developing future SNP chips.

Animals with difference in GBC also differed in their genomic architecture of quantitative traits, which was the case with 1,424 animals presented as Santa Gertrudis, and genomic prediction accuracy of these animals decreased as the GBC proportion of Santa Gertrudis decreased. Evidently, pooling animals with drastically differed GBC profiles could lower genomic prediction accuracies of validated (or purebred) animals. How to further improve genomic prediction of crossbred animals with estimated GBC remained to be explored in future studies.

Finally, estimation of GBC is conducted under the assumption that all the involving breeds contributed 100% to the genomic breed composition of each animal. This analysis, however, could be biased when there one or more ancestry breeds were missing in reality, regardless of which statistical models were used.

## Additional files

Additional file 1: **Figure S1.** Density plot of genomic breed composition of 1424 animals putatively presented as Santa Gertrudis cattle. (DOCX 23 kb)

Additional file 2: **Figure S2.** Map view of 1 K (A), 3 K (B), 5 K (C), 10 K (D) and 16 K (E) SNP panels, where 1 K, 3 K, 5 K and 10 L denotes 1000, 3000, 5000 and 10,000 SNP panels, respectively. The 1-10 K panels were obtained by maximizing average Euclidean distance of SNP allele frequencies among ten cattle breeds. The 16 K consisted of 15,708 common SNPs across five currently used bovine SNP chips. (DOCX 290 kb)

Additional file 3: **Figure S3.** Plot of -2logLikelihood values computed for 198 purported Akaushi cattle, based on the admixture model with 1 K versus 16 K SNP panels. (DOCX 25 kb)

Additional file 4: **Figure S4.** Plots of -2logLikelihood for 1770 reference Brangus animals (green circles) after removing outliers. The likelihood values were computed assuming the true allele frequencies of SNPs were equal to those of each of the 10 breeds, respectively. The assumed breeds (from left to right) are Brangus, Angus, Red Angus, Santa Gertrudis, Beef Master, Hereford, Holstein, Jersey, Wagyu, and Akaushi. (DOCX 37 kb)

Additional file 5: **Figure S5.** Plots of -2logLikelihood for 583 BeefMaster cattle after removing outliers. The likelihood values were computed assuming the true allele frequencies of SNPs were equal to those of each of the 10 breeds, respectively. The assumed breeds (from right to left) are Beef Master, Santa Gertrudis, Brangus, Hereford, Angus, Red Angus, Holstein, Jersey, Wagyu, and Akaushi. (DOCX 39 kb)

Additional file 6: **Figure S6.** Plots of -2logLikelihood for 291 Santa Gertrudis cattle after removing outliers. The likelihood values were computed assuming the true allele frequencies of SNPs were equal to those of each of the 10 breeds, respectively. The assumed breeds (from right to left) are Santa Gertrudis, Beef Master, Brangus, Red Angus, Angus, Hereford, Holstein, Wagyu, Akaushi, and Jersey. (DOCX 39 kb)

Additional file 7: **Table S1.** Correlations of -2logLikelihood values computed for 198 purported Akaushi cattle using the five SNP panels. **Table S2.** Genomic breed characterization of 198 purported Akaushi animals based on the admixture model using each of the five SNP panels. **Table S3.** Genomic breed characterization of 1424 purported Santa Gertrudis cattle based on the admixture model using the 1 K SNP panel. (DOCX 251 kb)

## Abbreviations

1 K: 1000 SNP panel for genomic breed composition estimation
10 K: 10,000 SNP panel for genomic breed composition estimation
16 K: 15,708 SNP panel for genomic breed composition estimation
3 K: 3000 SNP panel for genomic breed composition estimation
5 K: 5000 SNP panel for genomic breed composition estimation
AED: Average Euclidean distance
BFGS: Broyden-Fletcher-Goldfarb-Shanno method
BW: Birth weight
dEBV: De-regressed estimated breeding value
EPD: Expected progeny difference
FAT: Fat thickness
GBC: Genomic breed composition
GBCA: Genomic breed composition of Akaushi breed
GBCH: Genomic breed composition of Holstein breed
GBCSG: Genomic breed composition of Santa Gertrudis breed
GGP: GeneSeek genomic profiler
GPA: Genomic prediction accuracy
HCW: Hot carcass weight
IBD: Identical by descent
IBS: Identical by state
IQR: Interquartile range
LD: Linkage disequilibrium
LD: Low-density
LOOCV: Leave-one-out cross-validation
MAF: Minor allele frequency
MARB: Marbling score
MEBV: Molecular estimated breeding value
MWW: Maternal weaning weight
REA: Ribeye area
ROH: Runs of homozygosity
SC: Scrotal circumference
SD: Standard deviation
WW: Weaning weight
YW: Yearling weight
