# Supplementary figures and images for: Comparing SNP panels and statistical methods for estimating genomic breed composition of individual animals in ten cattle breeds
Source: BMC Genet. 2018 Aug 9;19:56. doi: 10.1186/s12863-018-0654-3 (PMC6085684; doi:10.1186/s12863-018-0654-3)

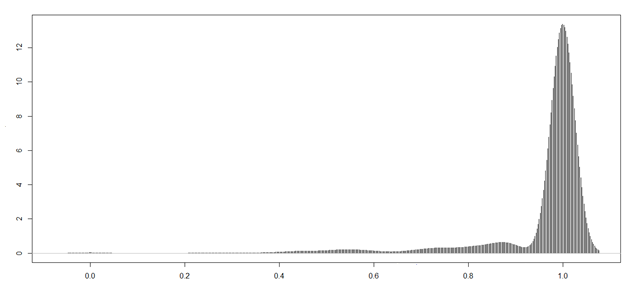

Supplement: Supplementary file 1 — Figure S1. Density plot of genomic breed composition of 1424 animals putatively presented as Santa Gertrudis cattle. (DOCX 23 kb) [file 12863_2018_654_MOESM1_ESM.docx]

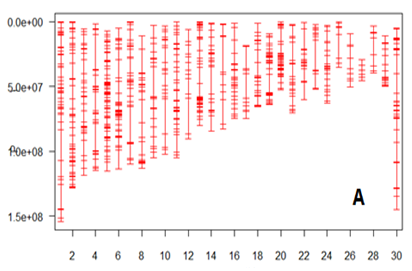

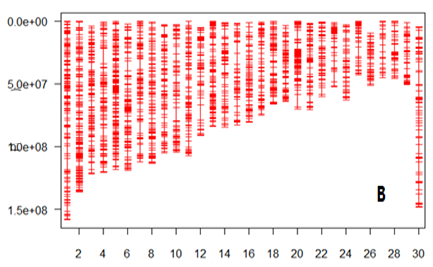


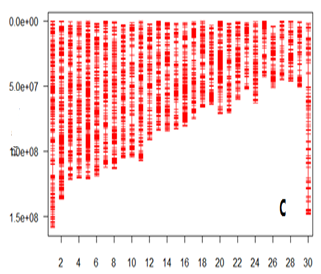

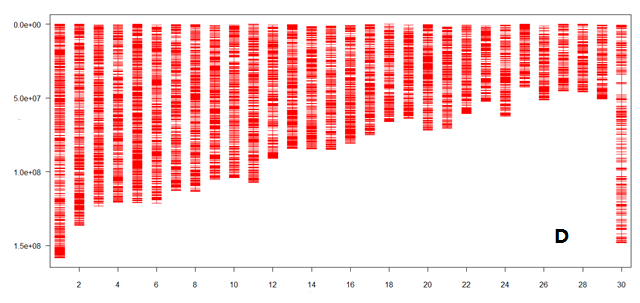

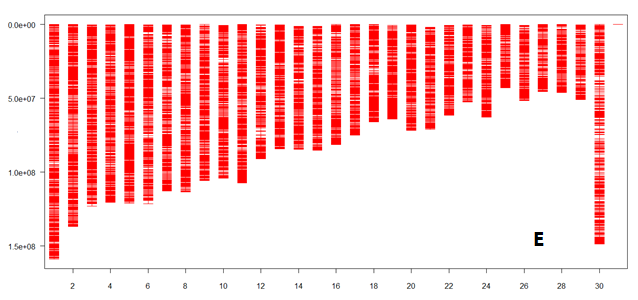

Supplement: Supplementary file 2 — Figure S2. Map view of 1 K (A), 3 K (B), 5 K (C), 10 K (D) and 16 K (E) SNP panels, where 1 K, 3 K, 5 K and 10 L denotes 1000, 3000, 5000 and 10,000 SNP panels, respectively. The 1-10 K panels were obtained by maximizing average Euclidean distance of SNP allele frequencies among ten cattle breeds. The 16 K consisted of 15,708 common SNPs across five currently used bovine SNP chips. (DOCX 290 kb) [file 12863_2018_654_MOESM2_ESM.docx]

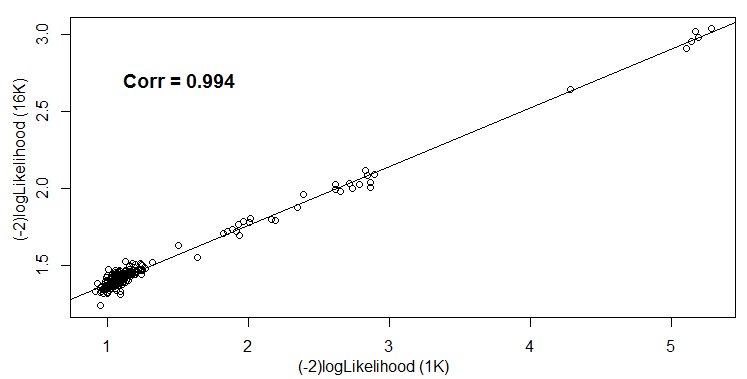

Supplement: Supplementary file 3 — Figure S3. Plot of -2logLikelihood values computed for 198 purported Akaushi cattle, based on the admixture model with 1 K versus 16 K SNP panels. (DOCX 25 kb) [file 12863_2018_654_MOESM3_ESM.docx]

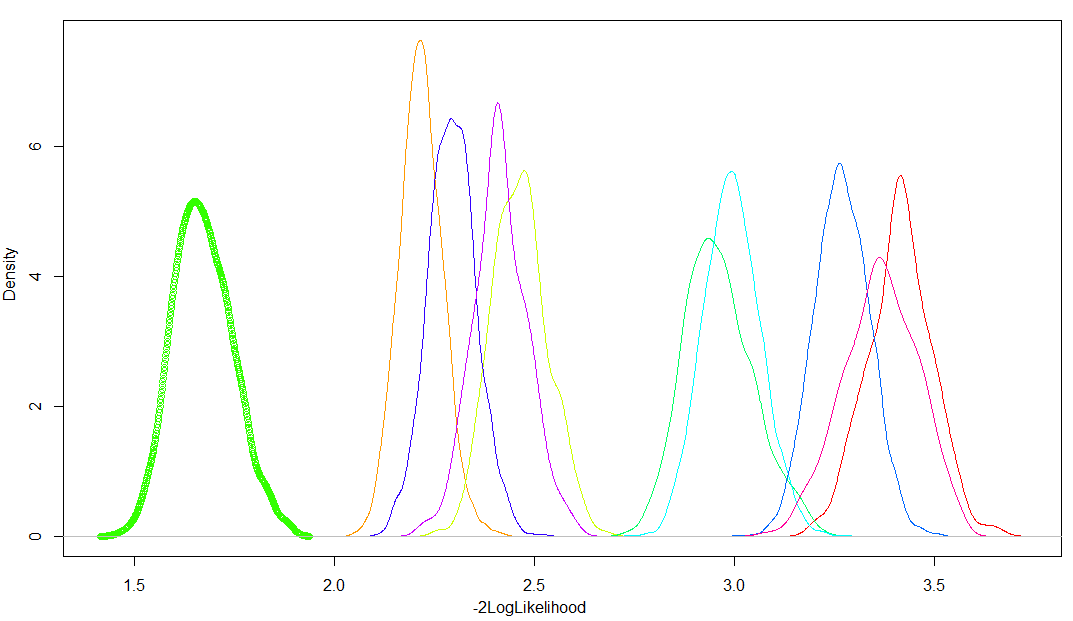

Supplement: Supplementary file 4 — Figure S4. Plots of -2logLikelihood for 1770 reference Brangus animals (green circles) after removing outliers. The likelihood values were computed assuming the true allele frequencies of SNPs were equal to those of each of the 10 breeds, respectively. The assumed breeds (from left to right) are Brangus, Angus, Red Angus, Santa Gertrudis, Beef Master, Hereford, Holstein, Jersey, Wagyu, and Akaushi. (DOCX 37 kb) [file 12863_2018_654_MOESM4_ESM.docx]

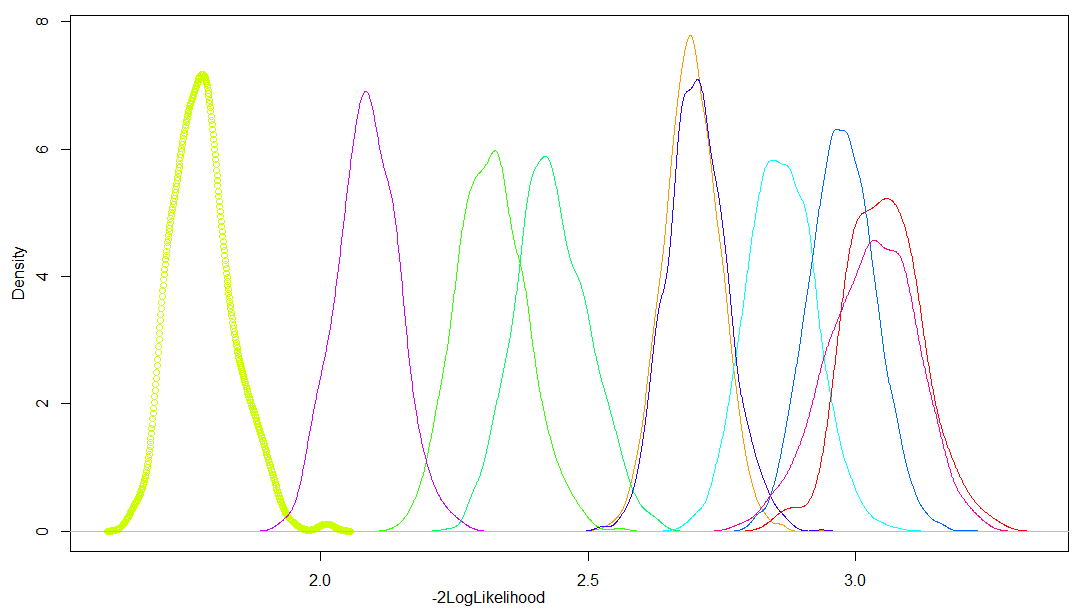

Supplement: Supplementary file 5 — Figure S5. Plots of -2logLikelihood for 583 BeefMaster cattle after removing outliers. The likelihood values were computed assuming the true allele frequencies of SNPs were equal to those of each of the 10 breeds, respectively. The assumed breeds (from right to left) are Beef Master, Santa Gertrudis, Brangus, Hereford, Angus, Red Angus, Holstein, Jersey, Wagyu, and Akaushi. (DOCX 39 kb) [file 12863_2018_654_MOESM5_ESM.docx]

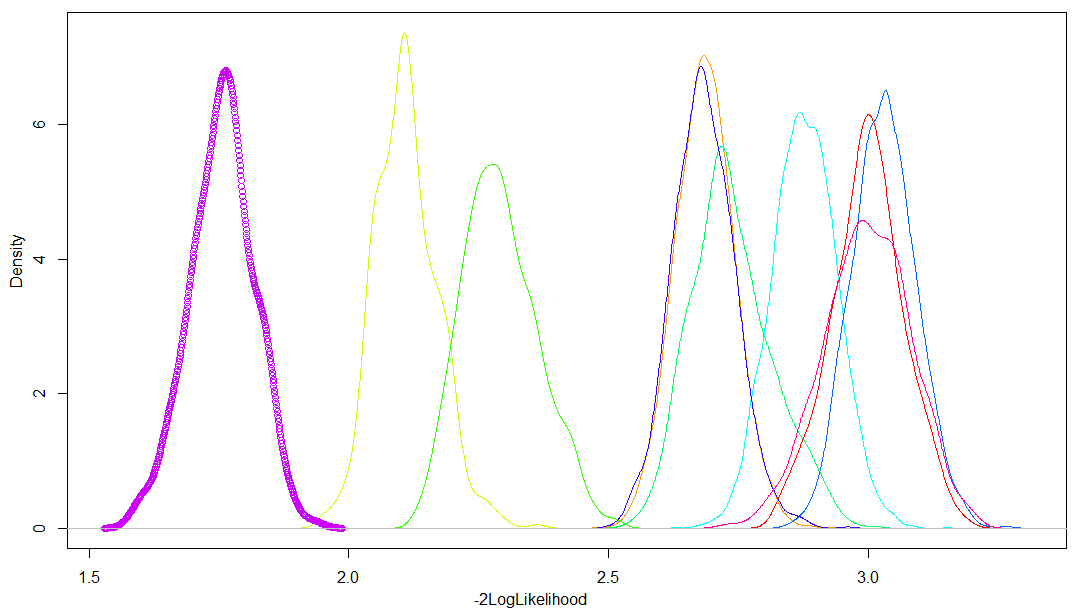

Supplement: Supplementary file 6 — Figure S6. Plots of -2logLikelihood for 291 Santa Gertrudis cattle after removing outliers. The likelihood values were computed assuming the true allele frequencies of SNPs were equal to those of each of the 10 breeds, respectively. The assumed breeds (from right to left) are Santa Gertrudis, Beef Master, Brangus, Red Angus, Angus, Hereford, Holstein, Wagyu, Akaushi, and Jersey. (DOCX 39 kb) [file 12863_2018_654_MOESM6_ESM.docx]
